# The Effect of Buffering High Acid Load Meal with Sodium Bicarbonate on Postprandial Glucose Metabolism in Humans—A Randomized Placebo-Controlled Study

**DOI:** 10.3390/nu9080861

**Published:** 2017-08-11

**Authors:** Pinar Kozan, Jackson C. Blythe, Jerry R. Greenfield, Dorit Samocha-Bonet

**Affiliations:** 1 Diabetes and Metabolism Division, Garvan Institute of Medical Research, 384 Victoria Street, Darlinghurst, NSW 2010, Australia; p.kozan@garvan.org.au (P.K.); jackson.blythe@outlook.com (J.C.B.); j.greenfield@garvan.org.au (J.R.G.); 2 St Vincent’s Clinical School, Faculty of Medicine, University of New South Wales, Level 5 deLacy Building, St Vincent’s Hospital, Victoria St., Darlinghurst, NSW 2010, Australia; 3 School of Medical Sciences, Faculty of Medicine, University of New South Wales, 18 High St., Kensington, Sydney, NSW 2052, Australia; 4 Department of Endocrinology, St. Vincent’s Hospital, 390 Victoria St., Darlinghurst, NSW 2010, Australia

**Keywords:** alkaline diet, dietary acid load, type 2 diabetes, acid-base homeostasis, sodium bicarbonate, postprandial glycaemia

## Abstract

**Background:** High dietary acid load relates to increased risk of type 2 diabetes in epidemiological studies. We aimed to investigate whether buffering a high acid load meal with an alkalizing treatment changes glucose metabolism post meal. **Methods:** Non-diabetic participants (*n* = 32) were randomized to receive either 1680 mg NaHCO_3_ or placebo, followed by a high acid load meal in a double-blind placebo-controlled crossover (1–4 weeks apart) study. Thirty (20 men) participants completed the study. Venous blood pH, serum bicarbonate, blood glucose, serum insulin, *C*-peptide, non-esterified fatty acid (NEFA), and plasma glucagon-like peptide-1 (GLP-1) concentrations were measured at baseline (fasting) and at 15–30 min intervals for 3 h post meal. **Results:** The treatment was well tolerated. Venous blood pH declined in the first 15 min post meal with the placebo (*p* = 0.001), but not with NaHCO_3_ (*p* = 0.86) and remained decreased with the placebo for 3 h (*p*_interaction_ = 0.04). On average over the 3 h blood pH iAUC was greater with NaHCO_3_ compared with placebo (*p* = 0.02). However, postprandial glucose, insulin, *C*-peptide, NEFA and GLP-1 were not different between treatments (*p*_interaction_ ≥ 0.07). **Conclusions:** An alkalizing medication administered pre-meal has no acute effect on glycaemia and insulin response in healthy individuals. Long-term interventions in at-risk populations are necessary to investigate the effect of sustained alkalization on glucose metabolism.

## 1. Introduction

The “Western diet”, characterized by a high intake of processed food, animal protein, and refined carbohydrates, combined with poor intake of fruit and vegetables, has become widespread in recent decades. Dietary protein from animal sources is rich in the sulphur-containing amino acids cysteine and methionine, which when metabolized lead to production of the non-volatile acids hydrochloric acid and hydrogen sulphate [1,2,3]. This process, coupled with a deficiency in the buffering of potassium and magnesium salts due to inadequate fruit and vegetable intake, renders the Western diet acidogenic in nature. It has been suggested that the high acid load of the Western diet contributes to the development of ‘mild metabolic acidosis’, a state of relatively low blood pH or serum bicarbonate concentration within the normal range [4]. 

Recent large cohort studies have suggested a link between high dietary acid load and insulin resistance or diabetes [5,6]. In these studies, the indices of diet acidity potential renal acid load (PRAL, based on the dietary intake of protein, phosphorous, potassium, magnesium and calcium) and net endogenous acid production (NEAP, based on the dietary intake of protein and potassium) were associated with insulin resistance [6] and diabetes [5,7]. However, other studies reported no association between dietary acid load indices and insulin resistance or diabetes [8,9]. The inconsistent findings in studies may relate to the different characteristics of the cohorts studied, and the inherent limitations associated with indices calculated from self-reported diet diaries.

In support of a link between body acidity and diabetes risk, a retrospective analysis of the US National Health and Nutrition Examination Survey found that lower serum bicarbonate concentrations were associated with insulin resistance, as measured by an index based on fasting serum insulin and triglycerides [10]. Longitudinal evidence from the Nurses’ Health Study showed that lower concentrations of plasma bicarbonate were associated with higher type 2 diabetes risk over 10 years [11]. 

Associations between acidosis and impaired glucose metabolism were originally identified almost a century ago [12], and early studies demonstrated that administration of ammonium chloride for three days to healthy individuals resulted in mild metabolic acidosis and increased insulin resistance [13]. Conversely, several studies reported that correcting overt metabolic acidosis in chronic renal failure patients with oral sodium bicarbonate improved insulin resistance [14,15].

Based on these findings, it has been suggested that adherence to a low PRAL (“alkaline”) diet or consumption of an alkaline supplement to offset the acid load of a meal may be used to improve glucose metabolism in at-risk individuals [16]. However, to have a chronic effect on glucose metabolism, the alkalizing effect of a low PRAL diet may, or may not, be mediated by acute (that is, immediately post-meal) mechanisms, relating to insulin secretion. Furthermore, it remains unclear whether an alkalizing intervention can affect postprandial glucose metabolism in individuals with normal renal function. In the present study, we performed a randomized double-blind placebo-controlled crossover study to determine the effect of pre-prandial oral sodium bicarbonate on postprandial glucose metabolism in non-diabetic participants with normal renal function. We hypothesized that buffering a high acid load meal will attenuate postprandial blood pH decrease and glycaemic response. 

## 2. Materials and Methods

The study was conducted according to the principles outlined in the declaration of Helsinki and the study protocol was approved by the St Vincent’s Hospital Human Research Ethics Committee (Sydney, Australia). All participants provided written informed consent prior to study commencement. The study was registered at clinicaltrials.gov (NCT02501343).

### 2.1. Participants

Participants were recruited between June 2015 and May 2016 from the general population by advertisements on noticeboards at the St Vincent’s Hospital precinct (Sydney, Australia) and the University of New South Wales (Sydney, Australia), and via social media and email distribution lists.

Prospective participants were screened via a telephone interview and if eligible, an in-person screening was performed by a physician (PK) at the Clinical Research Facility at the Garvan Institute of Medical Research (Sydney). Exclusion criteria included pregnancy and lactation, a personal history of diabetes, treatment with glucose lowering medication, fasting blood glucose > 7.0 mmol/L (126 mg/dL) and/or HbA1c > 6.5% (69 mmol/mol), and cardiovascular, respiratory, or inflammatory disease. Individuals treated with anti-hypertensive medications and medications known to affect glucose homeostasis were excluded. Individuals with serum creatinine > 90 μmol/L and/or eGFR < 60 mL/min/1.73 m^2^ or liver enzymes equal to or over twice the normal upper limit, current smokers, or excessive alcohol consumers (>20 g/day or 40 g/day for women and men, respectively) were excluded, as were individuals with unstable body weight in the three months preceding the study. 

**Figure 1:**
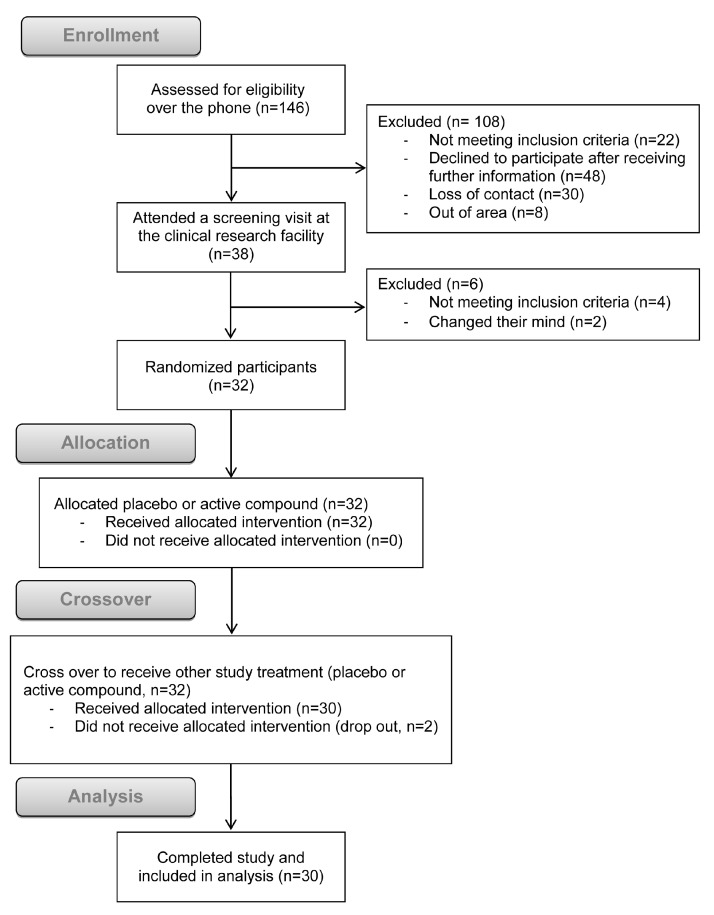
Consolidated Standards of Reporting Trials (CONSORT) flow diagram of the study (adapted from Schulz et al. [17]).

Participants responded to advertisements and were assessed for eligibility (*n* = 146). Following telephone and in-person screening, 114 participants were excluded. Thirty two participants (20 men) were randomized to receive the first treatment and two dropped out after the first study. Thirty participants (20 men) completed the crossover study and included in the analysis (Figure 1; study flow diagram). 

### 2.2. Study Design and Randomization

A randomized placebo-controlled double-blind crossover design was applied in a single centre, the Garvan Institute of Medical Research. Participants were randomized to receive an active treatment (1680 mg sodium bicarbonate; 2 × 840 mg Sodibic capsules, Aspen Australia, St Leonards, NSW, Australia) or identically looking placebo capsules (microcrystalline cellulose, Stenlake Compounding Chemist, Bondi Junction, NSW, Australia) 15 min prior to the first meal study. Participants returned for the second study, which was procedurally identical, but utilized the alternate treatment, 1–4 weeks after the first study. Participants and investigators were blinded to treatment allocation, with the randomization code (generated at www.randomizer.org) maintained securely by a nurse unrelated to the study. 

### 2.3. Meal Study Procedures

**Figure 2:**
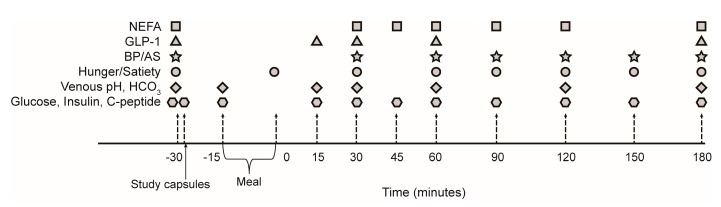
Overview of the meal study. The treatment was administered following collection of baseline (fasting) samples at *t* = −30 min. The sodium bicarbonate or placebo capsules were administered after collecting the baseline samples/measurements. Consumption of the meal commenced 15 min after administration of the treatment at *t* = −15, and participants allowed 20 min to complete the meal (*t* = 0 marks completion of the meal). Time points −15 and 15 min were only collected in *n* = 15 participants. Abbreviations: AS, arterial stiffness; BP, blood pressure; GLP-1, glucagon-like peptide 1; NEFA, non-esterified fatty acids.

Participants were asked not to exercise or drink alcohol for 48 h prior to the meal studies. To minimize variation between studies, participants were asked to follow a similar routine before the studies, with particular attention to the meal content on the night before the study. Participants reported to the Clinical Research Facility in the morning after an overnight fast, placed on a hospital bed, and an 18-gauge intravenous cannula inserted in the antecubital fossa. Baseline venous blood samples, blood pressure, arterial stiffness, and subjective satiety and hunger measures were ascertained at *t* = −30 min before administration of the intervention (Figure 2). The study capsules were administered 15 min prior to ingestion of a meal consisting of two breakfast muffins (Sausage and Egg McMuffin, McDonald’s Corporation^®^, Sydney, Australia) and 250 mL apple juice (Golden Circle^®^, Brisbane, Australia). Nutritional breakdown of the meal was calculated using FoodWorks 7 (Xyris, Spring Hill, QLD, Australia) based on the ingredients provided by the manufacturers (Table 1). 

Participants were given 20 min to consume the meal, with the 3 h follow up commencing at the completion of the meal (*t* = 0, Figure 2). Participants remained in bed in a reclined position for the following 3 h, with blood samples drawn and blood pressure, arterial stiffness and the hunger/satiety scores assessed periodically (Figure 2). 

### 2.4. Blood Pressure and Arterial Stiffness

Blood pressure was measured using a manual device (WelchAllyn, Arden, NC, USA) and arterial stiffness was measured by applanation tonometry of the radial artery with a highly-sensitive transducer (AtCor Medical, Sydney, Australia), as previously described [18]. The augmentation index (a surrogate of arterial stiffness) was calculated by the instrument’s program by dividing the difference between the second systolic peak and the diastolic pressure by the difference between the first systolic peak and the diastolic pressure (×100, %), as previously described [19].

### 2.5. Sample Collection and Analysis

Venous blood was collected and transported swiftly on ice and blood gases measured within 15 min of collection (Radiometer ABL 700 Blood Gas Analyzer, Diamond Diagnostics, Holliston, MA, USA). Serum electrolytes (sodium, potassium, bicarbonate, chloride, urea, and creatinine) were measured with the Roche Diagnostics Modular System (Indianapolis, IN, USA). 

Blood glucose was measured using YSI 2300 Stat Plus (YSI, Inc., Yellow Springs, OH, USA). Blood samples collected for insulin, *C*-peptide, non-esterified fatty acid (NEFA) and glucagon-like peptide-1 (GLP-1) analyses were immediately centrifuged (3500× *g* for 7 min at 4 °C), and serum (for insulin, *C*-peptide, and NEFA analyses) or plasma (for total GLP-1 analysis) snap frozen and stored at −80 °C until analysed at study completion. Serum insulin and *C*-peptide were measured using radioimmunoassay (Merck Millipore, St. Charles, MO, USA; intra-assay CVs were 4% and 7%, respectively) and NEFA concentrations using a colorimetric assay (Wako Diagnostics, Richmond, VA, USA; intra-assay CV 5%). Blood for GLP-1 analysis was collected into chilled 4 mL ethylenediaminetetraacetic acid (EDTA)-coated tube with dipeptidyl peptidase-4 inhibitor and trasylol to minimize GLP-1 degradation after collection. Total GLP-1 concentrations were analysed by radioimmunoassay (Merck Millipore, St. Charles, MO, USA) after extraction of plasma with 95% ethanol according to the manufacturer’s protocol (intra-assay CV 10%). 

### 2.6. Hunger and Satiety Assessment

Hunger and satiety were recorded using visual analogue scales. Briefly, participants were asked to rank on a scale of 1 to 10 how hungry they feel, with 0 representing “not hungry at all”, and 10 representing “most hungry”. Similarly, satiety was ranked with the question “how full do you feel?” with 0 representing not full at all and 10 representing the most possibly full. 

### 2.7. Statistical Analysis

A priori power analysis calculation was performed to detect a change in postprandial glucose excursions, measured by area under the curve of the glucose concentrations postprandially, based on a similar cohort studied by our group at the Clinical Research Facility at the Garvan Institute previously. Thirty participants were required to complete the study with α < 0.05 and statistical power 1 − β ≥ 0.8 (two-sided). All data are expressed as mean ± standard deviation (SD), unless when data were not normally distributed, in which data are presented as median and interquartile range (IQR). Data not normally distributed were logarithmically transformed prior to statistical analysis. Incremental area under the curve (iAUC) for the outcome measures was calculated using the trapezoidal rule [20]. Differences between iAUC of outcome measures post sodium bicarbonate vs. placebo were tested using paired *t*-tests. Two-way repeated measure ANOVA tests were conducted to assess differences in the response to the meal with sodium bicarbonate vs placebo, where *p*_time_ indicates the main effect of time (i.e., the meal on its own), *p*_treatment_ indicates the main effect of the treatment throughout the meal, and *p*_interaction_ indicates the effect of the interaction between time and treatment. Blood samples, blood pressure, arterial stiffness and hunger and satiety scores were collected at *t* = −30 min (baseline), then immediately after ingestion of the meal (0 min), then postprandially at 30, 45, 60, 90, 120, 150, and 180 min (Figure 2). A sub-cohort (*n* = 15) had additional blood sampled 15 min after administration of the treatment (*t* = −15) and 15 min post meal ingestion (*t* = 15; Figure 2). These time points were added after an interim examination of the blood pH data revealed that the predefined collection times (−30 and +30 min) may miss a rapid effect on venous blood pH. Data collected at −15 and 15 min were removed in the repeated measure ANOVA analyses, so that the tests were performed on the whole cohort (*n* = 30). One-sample *t*-test analyses were performed to assess the change in venous blood pH from baseline to 15 min (*n* = 15). Data were analysed using SPSS version 23 (International Business Machines, New York, NY, USA) and GraphPad Prism 6.07 (GraphPad Software, San Diego, CA, USA). 

## 3. Results

Participants (*n* = 30) were predominantly male (*n* = 20, 67%) and Caucasian (*n* = 24, 80%), with a median age of 31.5 (24.0–44.3) years. Participants tended towards the upper end of the normal weight range (24.0 (22.1–25.9)), with normal fasting blood glucose, serum insulin, and renal function, and fasting venous blood pH of 7.39 ± 0.02 (Table 2). All participants tolerated the sodium bicarbonate and no adverse events were noted.

### 3.1. Postprandial Acidity Markers with Sodium Bicarbonate or Placebo

**Figure 3:**
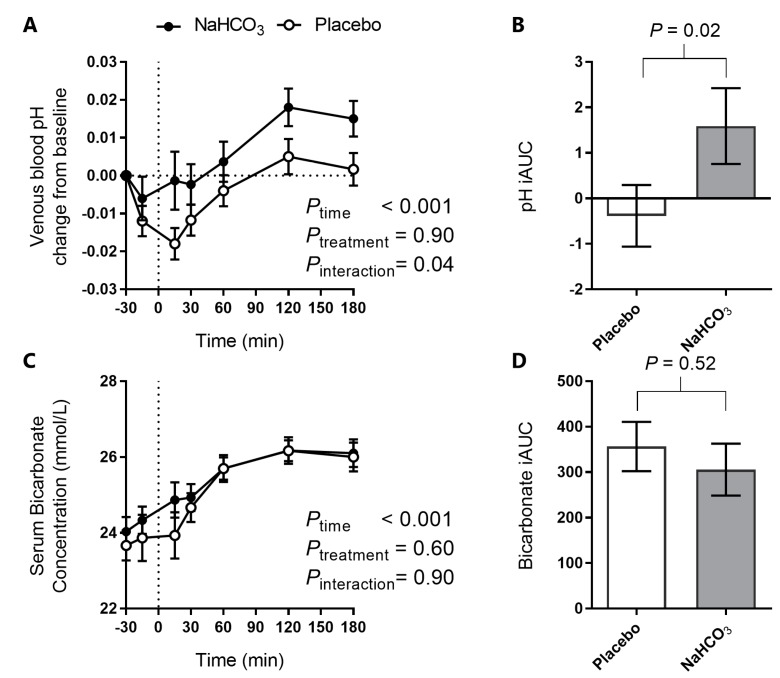
The effect of oral NaHCO_3_ (1680 mg) administered prior to a high acid load meal on venous blood pH (**A**); pH iAUC (**B**); serum bicarbonate (**C**); and bicarbonate iAUC (**D**), with NaHCO_3_ (solid circle) and placebo (hollow circle). Treatment was administered at *t* = −30 min and the meal completed at *t* = 0 (indicated by a dotted line). The effect of the treatment versus placebo on the postprandial outcome measure was tested by two-way repeated measure ANOVA and the difference in iAUC was tested by paired *t*-test, with *p* values indicated on the graphs. All data presented as mean ± standard error of the mean (SEM).

A significant postprandial effect on venous blood pH was noted with the meal (*p*_time_ < 0.001, Figure 3A), with an early decline in blood pH with the placebo, but not with the sodium bicarbonate. Specifically, a one-sample *t*-test at *t* = 15 min (*n* = 15) revealed that while venous blood pH under the placebo condition had decreased significantly from baseline (mean: 0.018, 95% CI: 0.009, 0.027; *p* = 0.001), this was not the case with the NaHCO_3_ treatment (mean: 0.001, 95% CI: −0.015, 0.018; *p* = 0.86). This finding is supported by a significant time-treatment interaction (*p*_interaction_ = 0.04, Figure 3A), and a difference in the overall pH iAUC between the placebo and bicarbonate treatment (Figure 3B). 

Serum bicarbonate increased and plateaued (Figure 3C) post-meal, without a significant difference between NaHCO_3_ and placebo, or significant difference in iAUC (Figure 3D). 

### 3.2. Postprandial Glucose Regulation with Sodium Bicarbonate or Placebo

**Figure 4:**
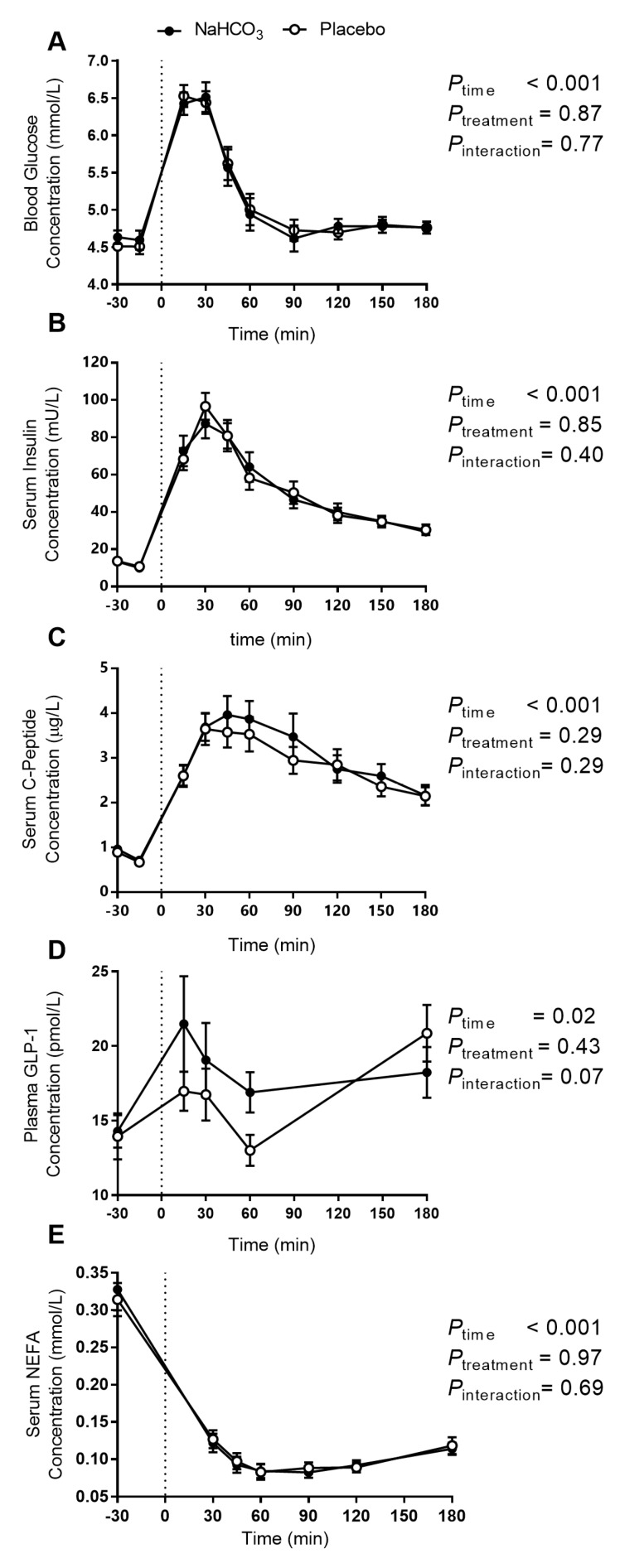
The effect of oral NaHCO_3_ (1680 mg) administered prior to a high acid load meal on blood glucose (**A**); serum insulin (**B**); serum *C*-peptide (**C**); plasma GLP-1 (**D**); and serum NEFA (**E**); comparing NaHCO_3_ (solid circle) and placebo (hollow circle). Treatment was administered at *t* = −30 min and the meal was completed at *t* = 0 (indicated by a dotted line). The effect of the treatment versus placebo on the postprandial outcome measure was tested by two-way repeated measure ANOVA, with *p*-values indicated on the graphs. All data presented as mean ± SEM. Abbreviations: GLP-1, glucagon-like peptide 1; NEFA, non-esterified fatty acids.

Blood glucose excursions (Figure 4A) were not different between the sodium bicarbonate treatment and placebo. A two-way repeated measure ANOVA test shows a significant main effect of the meal, but no treatment or time-treatment interaction (Figure 4A).

Similarly, no differences were observed in either insulin or *C*-peptide excursions with the meal (Figure 4B,C, respectively). Further, the ratios of insulin-to-*C*-peptide, glucose-to-insulin, and glucose-to-*C*-peptide, indices of insulin secretion and clearance, were calculated at each time point (data not shown). No significant differences were observed in two-way repeated measure ANOVA tests (*p*_interaction_ ≥ 0.11, other than the main effect of the meal, which was significant (*p* < 0.001) in all cases).

Two-way repeated measure ANOVA test of plasma GLP-1 concentrations revealed a significant time effect and a trending time-treatment interaction (*p* = 0.07; Figure 4D), with a non-significant difference in the overall GLP-1 iAUC between treatments (*p* = 0.43, data not shown). 

Serum NEFA concentrations were suppressed post meal, without a significant difference between bicarbonate and placebo (Figure 4E). 

### 3.3. Satiety and Hunger with Sodium Bicarbonate or Placebo

**Figure 5:**
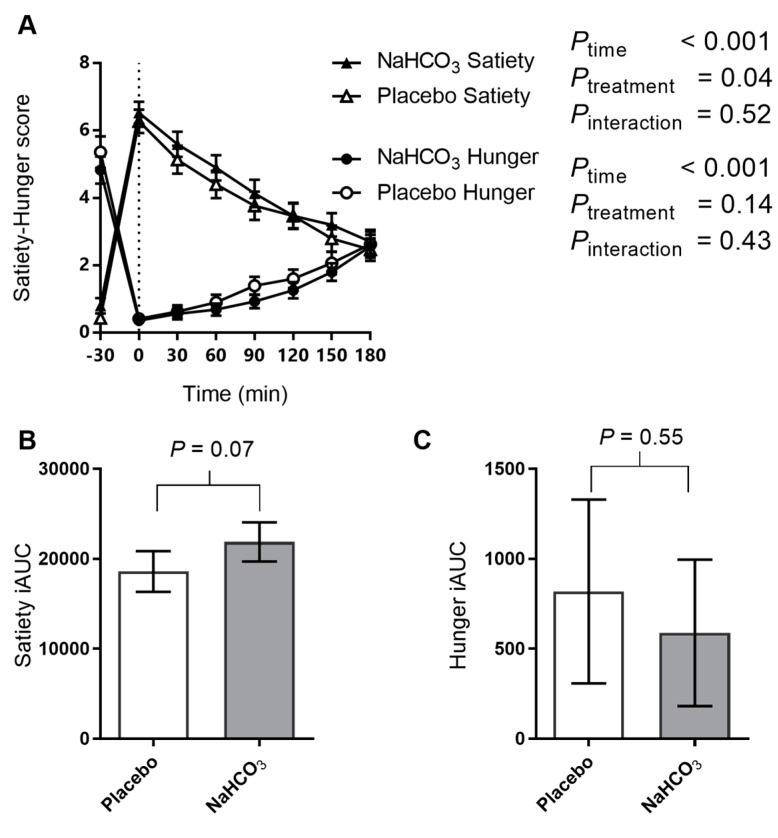
The effect of oral NaHCO_3_ (1680 mg) administered prior to a high acid load meal on satiety and hunger scores (**A**); satiety iAUC (**B**); and hunger iAUC (**C**); comparing NaHCO_3_ (solid symbol) and placebo (hollow symbol). Treatment was administered at *t* = −30 min and the meal was completed at *t* = 0 (indicated by a dotted line). The effect of the treatment versus placebo on the postprandial outcome measure was tested by two-way repeated measure ANOVA and the difference in iAUC was tested by paired *t*-test, with *p*-values indicated on the graphs. All data presented as mean ± SEM.

Satiety and hunger were affected by the meal (Figure 5A), and while satiety iAUC tended to increase with the bicarbonate treatment (Figure 5B), hunger was unaffected by the treatment (Figure 5C). 

### 3.4. Arterial Stiffness and Blood Pressure with Sodium Bicarbonate or Placebo

**Figure 6:**
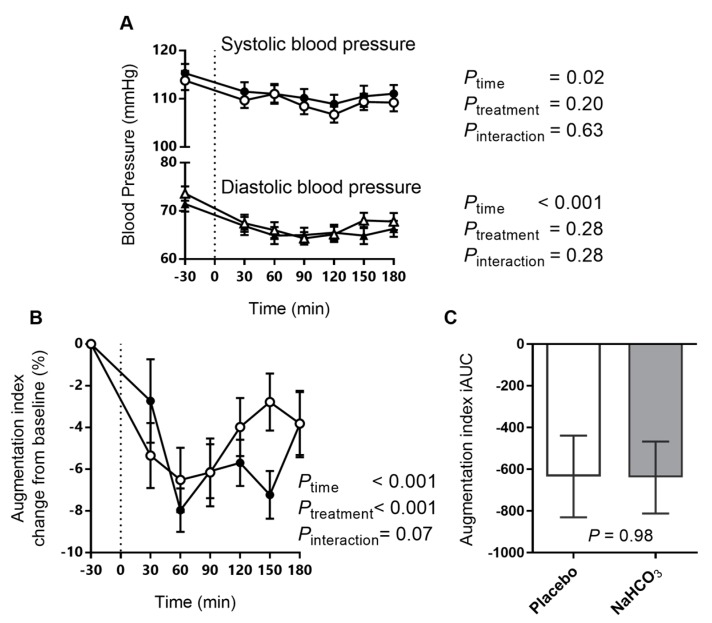
The effect of oral NaHCO_3_ (1680 mg) administered prior to a high acid load meal on systolic and diastolic blood pressure (**A**); the augmentation index (**B**) and the augmentation index iAUC (**C**), comparing NaHCO_3_ (solid symbol) and placebo (hollow symbol). Treatment was administered at *t* = −30 min and the meal was completed at *t* = 0 (indicated by a dotted line). The effect of the treatment versus placebo on the postprandial outcome measure was tested by two-way repeated measure ANOVA and the difference in iAUC was tested by paired *t*-test, with *p* values indicated on the graphs. All data presented as mean ± SEM.

Blood pressure decreased with the meal (Figure 6A), but both the systolic and diastolic blood pressure postprandial responses were unaffected by the bicarbonate treatment. 

Arterial stiffness, measured by the augmentation index, decreased with the meal (Figure 6B). Two-way repeated measure ANOVA test revealed significant main effects of time and treatment on the augmentation index, with a tendency to treatment-time interaction (Figure 6B). However, no difference in the overall augmentation index iAUC between treatments was observed (Figure 6C).

## 4. Discussion

This study reports that sodium bicarbonate treatment attenuates the post-meal decrease in pH observed with placebo in a cohort of non-diabetic individuals with normal renal function. Despite the attenuation in the pH decline achieved with sodium bicarbonate, no effect on postprandial glucose homeostasis was found. Interestingly, while it has previously been proposed that a high acid load diet decreased blood pH [21], a search of the literature indicates that the present study is the first to demonstrate and quantify this phenomenon in the context of a meal tolerance test.

Postprandial insulin and *C*-peptide excursions were not different between the bicarbonate treatment and placebo, consistent with the overlapping curves observed for glucose excursions. Together, these findings indicate that there was no acute effect of the alkalizing treatment on insulin secretion. A non-significant increase in plasma GLP-1 excursion with the alkalizing treatment was identified. However, the GLP-1 rise did not elicit concomitant increases in insulin. Together with the trending effect of the alkalizing treatment on satiety, these findings may suggest slower gastric emptying with the treatment. However, this was not tested in the present study. The long term implications of postprandial circulating GLP-1 enhancement with bicarbonate and the mechanism(s) involved in this response require further investigation.

Several explanations to the lack of glycaemic effects with bicarbonate treatment observed here may be suggested. The first, and perhaps the best supported, would be that the pH change induced by the pre-prandial oral bicarbonate was not potent enough to elicit meaningful changes in glucose metabolism. This may be due to either the relatively low dose of bicarbonate employed or the potent buffering capacity of the body overcoming exogenous bicarbonate in healthy individuals. While more substantial venous blood pH increases have been documented with higher oral NaHCO_3_ doses (0.3 g/kg body weight [22], approximately 10-fold the dose administered here), such doses are associated with severe gastrointestinal side effects [22,23], rendering any potential clinical application impractical. In any case, although the pH change induced in the present study is small, it closely reflects the degree of venous alkalization reported after a four-day adherence to low-PRAL (alkaline) diet [24]. 

The alkaline diet is intended to remedy the excessive acid load of the Western diet, with many proposed health benefits. Advocates of the alkaline diet suggest that by increasing body pH (measured for example by increasing urine pH), individuals reduce their risk of conditions ranging from cancer to osteoporosis [25]. Many of these claims have been challenged recently [26,27], with most of the observed benefit of the alkaline diet possibly stemming from increased fruit and vegetable intake and the associated increase in dietary potassium-to-sodium ratio [25]. With regards to insulin resistance and type 2 diabetes, some body of evidence suggests that adherence to an alkaline diet may be protective [5,6,28]. The acute nature of the present study excludes the investigation of the long term effects of venous pH alkalization on whole body insulin resistance. However, our findings exclude an acute effect of body alkalization on glycaemia and insulin secretion, at least in healthy individuals. 

Following a meal, arterial stiffness decreases as a component of the body’s response to insulin, a drop that is attenuated in insulin resistance [18]. In the present study, arterial stiffness tended to decrease after the bicarbonate treatment compared with placebo. Similarly, bicarbonate is known to decrease arterial stiffness when added to haemodialysis fluids [29], however, the mechanism for this phenomenon has not been elucidated and requires further study. 

The strengths of the present study are the robust randomized crossover placebo-controlled design and the direct measurements of blood pH and glucose metabolism in a high acid load meal context. However, the present study has some limitations. Firstly, this study did not target insulin-resistant individuals and, therefore, the findings can only be applicable to a relatively healthy population. While several studies have reported improvement in insulin sensitivity after correcting metabolic acidosis in chronic renal failure patients [14,15], reparation of an abnormally low pH may be physiologically different to increasing pH within the physiological range in healthy individuals with respect to its impact on glucose metabolism. Additionally, this study only examined the acute effect of a relatively low dose bicarbonate supplementation on glucose metabolism. The effect on insulin resistance cannot be studied in the acute settings and, therefore, a chronic intervention is necessary to establish whether an alkalizing treatment will correct glucose metabolism in individuals with pre-diabetes. Furthermore, a low-acid load meal comparator was not tested, and finally, while increased venous blood pH was detected with the bicarbonate treatment, there was no detectable change in serum bicarbonate. While this was unexpected, it may be related to the sensitivity of the serum bicarbonate assay. 

Importantly, the present study indicates that the beneficial glycaemic effects documented in populations adhering to low acid load diets are not likely to be explained by an acute effect on postprandial glycaemia. Further studies are required to elucidate the long term effects of buffering high acid load diets on type 2 diabetes risk and to shed light on the dynamics of body acid-base balance and glucose homeostasis.

## Figures and Tables

**Table 1:** Nutritional breakdown of the study meal.

Constituent	Average Per Meal
Energy (kJ)	3567
Protein (g)	42
Fat (g)	39
Saturated fat (g)	18
Total carbohydrates (g)	79
Sugar (g)	31
Sodium (g)	1.3
^a^ PRAL (mEq)	18.3

Based on information provided by McDonalds^®^ and Golden Circle^®^. ^a^ PRAL (potential renal acid load) is calculated from dietary intake as follows, PRAL (mEq) = 0.49 × Protein (g) + 0.037 × Phosphorous (mg) − 0.021 × Potassium (mg) − 0.026 × Magnesium (mg) − 0.013 × Calcium (mg).

**Table 2:** Characteristics of the study cohort.

*N* (Men)	30 (20)
Age (years)	31.5 (24.0–44.3)
Waist circumference (cm)	83.0 (77.0–91.0)
Weight (kg)	74.4 ± 14.9
BMI (kg/m^2^)	24.0 (22.1–25.9)
Family history of type 2 diabetes (*n*)	10
Systolic blood pressure (mmHg)	120 ± 12
Diastolic blood pressure (mmHg)	77 ± 7
^a^ Fasting blood glucose (mmol/L)	4.6 ± 0.4
^a^ Fasting serum insulin (mU/L)	10.8 (8.0–17.3)
^a^ HOMA-IR	2.3 (1.7–3.6)
Serum creatinine (μmol/L)	81.1 ± 10.1
^a^ Fasting venous pH	7.39 ± 0.02

Data expressed as mean ± standard deviation (SD) or median with interquartile range (IQR, for data not normally distributed). ^a^ Average of measurements performed on two separate days; HOMA-IR, homeostatic model assessment of insulin resistance was calculated as follows, Fasting blood glucose (mmol/L) × Fasting serum insulin (mU/L)/22.5.
